# Integrated Implementation of Programs Targeting Neglected Tropical Diseases through Preventive Chemotherapy: Proving the Feasibility at National Scale

**DOI:** 10.4269/ajtmh.2011.10-0411

**Published:** 2011-01-05

**Authors:** Mary Linehan, Christy Hanson, Angela Weaver, Margaret Baker, Achille Kabore, Kathryn L. Zoerhoff, Dieudonne Sankara, Scott Torres, Eric A. Ottesen

**Affiliations:** RTI International, Washington, District of Columbia; United States Agency for International Development, Washington, District of Columbia; Department of International Health, School of Nursing and Health Sciences, Georgetown University, Washington, District of Columbia; Liverpool Associates in Tropical Health, Washington, District of Columbia

## Abstract

In 2006, the United States Agency for International Development established the Neglected Tropical Disease (NTD) Control Program to facilitate integration of national programs targeting elimination or control of lymphatic filariasis, onchocerciasis, schistosomiasis, soil-transmitted helminthiasis and blinding trachoma. By the end of year 3, 12 countries were supported by this program that focused first on disease mapping where needed, and then on initiating or expanding disease-specific programs in a coordinated/integrated fashion. The number of persons reached each year increased progressively, with a cumulative total during the first three years of 98 million persons receiving 222 million treatments with donated drugs valued at more than $1.4 billion. Geographic coverage increased substantially for all these infections, and the program has supported training of more than 220,000 persons to implement the programs. This current experience of the NTD Control Program demonstrates clearly that an integrated approach to control or eliminate these five neglected diseases can be effective at full national scale.

## Introduction

The neglected tropical diseases (NTDs) are a group of conditions causing significant morbidity and mortality worldwide but which until recently received only minimal attention from most of the world, largely because they affect the poorest, most vulnerable and most disenfranchised members of society.^1^ Afflicting more than one billion persons, one-sixth of the world's population, these diseases cause severe disfigurement, disability, and blindness. The NTDs are among the leading perpetuators of poverty because they significantly diminish economic productivity in affected adults and because they impair the intellectual and physical development of the next generation in disease-endemic areas, setting already vulnerable children on a path to lifelong disability that reinforces a cycle of poverty.^1^

Among the 15 most prominent NTDs,^2^ seven have similar strategies to address their control; namely, single doses of effective treatment, termed preventive chemotherapy (PCT), given once or twice a year to broad segments of the population in disease-endemic areas through mass drug administration (MDA). These seven diseases are lymphatic filariasis (LF), onchocerciasis, schistosomisis, trachoma, and three soil-transmitted helminth (STH) infections (ascariasis, hookworm, and trichuriasis).^3^ The treatment and diagnostic tools currently available for this group of diseases are sufficiently effective for these NTDs to be targeted either for elimination or for reduction to such low levels that they no longer constitute a significant public health problem.

Most of the drugs used in these single-dose, once- or twice a year treatment regimens are donated through large public-private partnerships that bring together public health implementers, public-sector and private-sector donors, and the major pharmaceutical firms producing these drugs (GlaxoSmithKline, Johnson & Johnson, Merck and Co., Inc., Merck-Serono, and Pfizer).^4^

Historically, many Ministries of Health in disease-endemic countries have supported the control of NTDs through independent, often parallel, programs, with each maintaining its own planning, funding, drug supply chain, MDA campaign, monitoring, and evaluation. If funding were available for one program, that program might have been able to implement preventive chemotherapy while a sister program could not. However, because there is considerable overlap of these diseases in persons and communities, controlling one of the NTDs and not others that could be managed through a similar strategy is inefficient at best. Furthermore, research has provided sufficient evidence to suggest that co-implementation of the integrated PCT programs is safe for persons and communities in all but a few specific settings (e.g., *Loa loa* co-endemicity with onchocerciasis or LF).^3^

Because of the similarity of their strategic approaches, the epidemiologic overlap among affected populations and the availability of donated drugs, these NTD control programs seemed ideally suited for implementation that could be carried out not in parallel, independent fashion, but, rather integrated in a way where coordinated treatment interventions for multiple diseases could reduce the duplication of effort expended in treating the diseases separately. Such integration, here considered in the broadest sense as coordination of program activities among different disease-specific programs and as linkages of these activities with other elements of the health care system, should lead to efficiencies of delivery, enhanced effectiveness, increased health benefits, and better use of limited resources that could permit more at-risk persons to be reached.^5^ The World Health Organization (WHO) has endorsed such co-implementation of programs as the integrated approach to preventive chemotherapy.

Early pilot studies of NTD program integration generally showed that despite many practical challenges, such integration was likely to be feasible and to result in at least some of the anticipated efficiencies and cost savings.^6^–^8^ Although it was clear from these pilot studies that certain elements of program implementation were more amenable to successful integration than others, it was not clear either how successful the scaling up of these integration efforts from pilot studies to national-scale programs could be or just which combinations of activities were most effectively linked.

The opportunity to document the feasibility of integrated approaches to NTD control at full national-scale presented itself when the U.S. Congress in 2006 authorized funds for “the integrated control of neglected tropical diseases.”^9^ This authorization led to the establishment of NTD Control Program of the United States Agency for International Development (USAID) that envisioned, over a five-year period, facilitating integrated NTD programs in 15 countries. The present report documents the considerable achievements of the first three years of this NTD Control Program towards the development and growth of national integrated NTD programs and in their expansion to full national scale.

## Methods

### The NTD Control Program.

#### Goals and approach.

The USAID NTD Control Program initiated activities in September 2006. Its defined target was to enable the provision of 160 million preventive chemotherapy treatments to 40 million people in 15 countries through integrated NTD programs over five years. The stated aims of the program have been 1) to support and empower national governments to develop integrated NTD control programs embedded, where possible, within existing service delivery platforms and to lead these programs in scaling-up activities to full national levels; 2) to provide technical assistance for planning, budgeting, reporting, and complying with international standards and guidelines (Table 1) to improve program integration; 3) to promote cost-efficiency, improved integration strategies, and effective advocacy; and 4) to assure national ownership, continued commitment, and resource mobilization for sustained support for NTD control.

The prime contractor, RTI International, has provided grants and coordination for a team of non-governmental organizations (NGOs) and implementing partners to support integrated NTD control programs organized and led by the governments of selected countries. The support from the U.S. Government was intended to build on existing commitments by governments and other donors and fill financial and technical gaps that were preventing national programs from reaching full national scale. The program was mandated to track and report on the additional numbers of persons reached and treatments provided through support of the NTD Control Program (recognizing that in some countries other support also exists for NTD control).

### Participating countries and NGOs.

The countries currently involved in the USAID-supported, RTI-coordinated NTD Control Program are identified in Table 2, along with the lead NGO responsible in each country for interfacing between the national Ministry of Health and RTI. Those five countries that had earlier pilot programs, initiated with support from the Bill and Melinda Gates Foundation^10^ and aimed at integrating disease-specific NTD control activities, are referred to as the fast-track countries because they could begin scaling up activities immediately; additional countries have been brought into the program progressively. By the end of year 3, 12 countries were included in the program, of which 7 were actively engaged in yearly MDAs. It is from these first seven implementing countries that the quantitative measures of the NTD Control Program's programmatic achievement during its first 3 years (reported below) are derived.

### Drug distribution.

All drugs were distributed by the national Ministries of Health whose national NTD control programs determined how best to implement their MDAs (Table 2). The drugs were used according to WHO recommendations (Table 1). When disease co-endemicity required the use of multiple drugs (including combinations of albendazole, diethylcarbamazine [DEC], ivermectin, mebendazole or praziquantel), these were generally given at the same time, although sometimes the praziquantel treatment was delayed for at least a week after the other drugs were administered. When azithromycin was required, its administration was always at least a week separated from those of the other drugs, as currently recommended.

Albendazole for treatment of LF was donated by GlaxoSmithKline^11^; when used to treat STH in areas where LF is not endemic, albendazole was obtained from pre-qualified generic manufacturers. Azithromycin (Zithromax) was donated by Pfizer.^12^ Diethylcarbamazine was obtained through WHO from pre-qualified generic manufacturers. Ivermectin (Mectizan) was donated by Merck & Co., Inc.^13^ Mebendazole was donated by Johnson & Johnson^4^ to treat persons with STH in countries where its Children Without Worms program operates. Tetracycline eye ointment was obtained from pre-qualified generic manufacturers.

### Technical assessments.

#### Mapping.

Because knowledge of the distribution of each NTD in a country is absolutely essential for developing any implementation (or integrated-implementation) plan, disease-specific mapping was carried out according to guidelines recommended by WHO and its partners (Table 3). Although some of these guidelines are still evolving, for all program assessments, the most up-to-date recommendations were followed.

#### Program metrics.

To document progress toward achieving the program's targets of 40 million additional persons treated with 160 million treatments over 5 years, the following indicators were used to track country-specific program progress: 1) number of countries supported by the NTD Control Program; 2) number of additional districts (implementation units) mapped for each endemic disease; 3) number of people treated (i.e., receiving at least one drug or drug package) and recorded in MDA registers for each round of PCT; 4) number of additional (i.e., made possible through support from the NTD Control Program) treatments provided (i.e. number of times a single drug dose is administered) and recorded in MDA registers for each round of PCT; 5) number of additional implementation units reached and reported by national programs for each round of PCT; 6) programmatic coverage: % targeted population reached with appropriate PCT treatment each round of PCT and calculated from register reports as the number treated divided by treatment-eligible population targeted in the implementation unit (as defined by census reports); 7) geographic coverage: % of endemic districts covered by PCT programs; and 8) number of persons trained for integrated NTD control through support from the NTD Control Program.

### Program expenditures.

Funding levels for each of the program's first three years approximated $13.5 million per year, with the mandate that at least 80% of total annual resources be allocated to country program implementation and that overall management costs by RTI (whose role was to ensure financial accountability of all funds expended and to provide requested technical assistance to national programs) be no more than 20%. Of the 80% allocated for country implementation activities, approximately 20% was earmarked for procurement of essential drugs not available through donation programs (i.e., PZQ for schistosomiasis, DEC for LF, and albendazole for childhood de-worming in areas where LF is not co-endemic).

## Results

### Mapping the geographic distribution of the targeted NTDs.

Because knowing the distribution of the targeted NTDs is essential for developing an implementation strategy, the first efforts of the NTD Control Program in the participating countries focused on cataloging the disease-distribution information available and then supporting on-the-ground efforts to map the distribution of infection where sufficient information was not available.

Table 4 aggregates the data from all of the districts in the first seven implementing countries (identified in Table 2) and indicates the total number of districts that had been mapped for each of the NTDs prior to the initiation of the NTD Control Program. It is clear that mapping was already well advanced for LF. For the other diseases, there was still a great need for additional information on the distribution of these infections.

Table 4 also shows the progress made by the end of the first three years of support to the participating countries through the NTD Control Program. For each of the targeted diseases, mapping activities progressively defined the extent of the targeted diseases, and as a result of these efforts and those of other partners, only a small number of districts in these countries remained to be mapped for these NTDs at the end of year 3 (now targeted for subsequent years' activities).

### Persons treated and number of treatments delivered in years 1–3.

**Figure 1.**
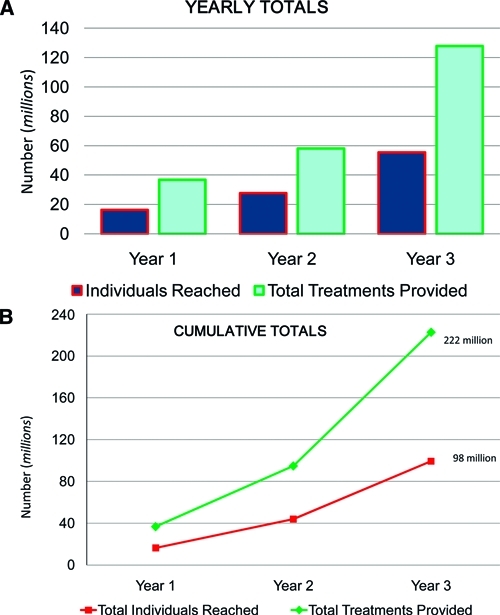
**A**, Persons reached (dark bars) and treatments provided (light bars) during each of the first three years of the Neglected Tropical Disease (NTD) Control Program. **B**, Cumulative totals of persons reached (dark line) and treatments provided (light line) over the first three years of the NTD Control Program.

Although programs targeting the individual NTDs were active in many countries prior to the inception of the NTD Control Program, few countries approached these diseases with an integrated control strategy. As seen in Figure 1A, after the fast track countries, with earlier support for pilot-scale integration studies by the Bill and Melinda Gates Foundation,^10^ began to receive support for integrated programs from the NTD Control Program, there was a progressive increase in numbers of persons reached each year, beginning with 16 million additional individuals in the first year and reaching 27 and 55 million additional persons in each of the second and third years. (These numbers identify only those persons whose treatment was made possible by support to national programs from the NTD Control Program, cumulatively more than 98 million persons over three years.)

Because a treated person in these integrated programs often receives a drug package comprising more than one medication, the metric ‘treatments provided' was developed to record the number of individual drug treatments received by the target population. As indicated in Figure 1B, 222 million drug treatments were provided during the first three years of support to the 7 implementing countries by the NTD Control Program. The details of these treatments provided (drugs treatments distributed by the seven participating national programs) are shown in Table 5.

### Quantity/value of drugs delivered.

As the national programs supported by NTD Control Program funds increased in number and expanded in scope, the number of donated tablets of drug delivered to the national NTD programs has also increased. Table 6 shows that in year 3 alone more than 300 million drug tablets were donated and delivered to the countries receiving NTD Control Program support. The value of these drugs (as defined by each specific donation program) exceeded $575 million dollars in year 3 and has totaled more than $1.4 billion dollars in the program's first three years.

### Coverage.

The *sine qua non* for success of preventive chemotherapy programs is high rates of drug coverage in the disease-endemic populations. Although varying among countries and for specific programs within each country, programmatic coverage (the percentage of targeted persons who actually received the drug) was generally good (Table 7). These values were based on numbers reported by the drug distributors and their supervisors; and when these reported values were subjected to validation studies in coverage surveys, there was generally good agreement between the reported and surveyed coverage values (data not shown).

**Figure 2.**
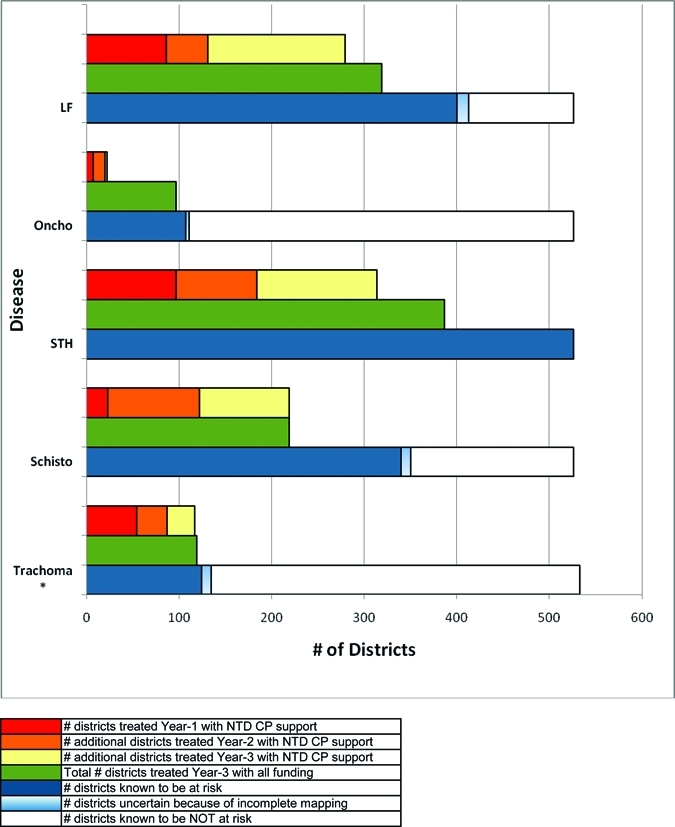
Number of districts covered by mass drug administration (MDA) treatment during the first three years of the Neglected Tropical Disease (NTD) Control Program in the seven implementing countries (an aggregated total of 526 districts in these countries). For each of the diseases targeted, the bottom bar depicts the number of districts known to be at risk (dark blue bar), the number known not to be at risk (white bar), and those where uncertainty remains because of incomplete mapping (light blue bar). For each of the diseases, the top bar represents the number of districts implementing MDA with the United States Agency for International Development NTD Control Program support (the red bars indicate the number supported in the first year, the orange bar indicates the additional numbers supported in the second year, and the yellow bar indicates the additional supported in the third year). For each disease, the middle bar (green) indicates the total number of districts receiving MDA treatment supported by any funding source. LF = lymphatic filariasis; Oncho = onchocerciasis; STH = soil-transmitted helminths; Schisto = schistosomiasis. *Ghana interrupted transmission of trachoma during year 2 and therefore did not require treatment in year 3.

For successful elimination and large-scale control programs using the preventive chemotherapy strategy, it is also necessary to have broad geographic coverage (the percentage of disease-endemic districts covered by PCT programs). Figure 2 records the numbers of districts under treatment of each disease during the first 3 years. It shows that for each of the NTDs, the geographic coverage increased progressively during the three years of NTD Control Program activity. Because the program can only expand (i.e., increase geographic coverage) in areas where mapping is complete, those NTDs where mapping is more advanced (e.g., onchocerciasis where the African Program for Onchocerciasis Control has been a strong and consistent source of funding for mapping and implementation) have the greatest geographic coverage. By the end of year 3, geographic coverage in the 7 implementing countries had increased for each of the NTDs, ranging from a high of 95% for onchocerciasis to a low of 50% for schistosomiasis. The treatment gap remaining for each disease in the first seven countries and targeted in the upcoming years can be appreciated in Figure 2.

### Training/national capacity building.

**Figure 3.**
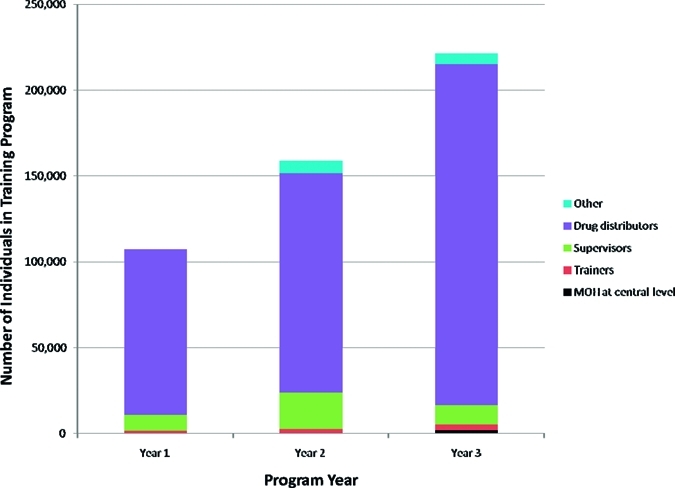
Number of workers in training programs supported by the Neglected Tropical Disease Control Program. For each of the first three years of the program, the number of persons receiving different types of training are recorded (black indicates training for central-level Ministry of Health [MOH], orange indicates training for trainers, green indicates training for supervisors, purple indicates training for drug distributers, and blue indicates training for others).

At the heart of all PCT programs is the community that is affected by NTDs. Training is designed to empower these communities to treat NTDs within their own populations. The NTD Control Program has supported the training of more than 220,000 persons during its first three years, with most being community-based health workers/drug distributors (Figure 3). Working at levels from the central ministries to the communities, a cascade of training has been facilitated to support social mobilization, community outreach, supply chain organization and management, and technical implementation of PCT. The fundamental content of training courses and refresher training is similar for most programs, but local needs dictate local training and organizational strategies.

### NTD Control Program expenditures.

Analysis of expenditures during the first three years shows that the program fulfilled its mandate that at least 80% of program funds be spent on country program implementation. During the first three years, the program received $40,728,320 in funding. Program expenditures by the end of year 3 were $37.9 ($2.8 million remaining to be spent), with 81.3% expended for country program activities and purchase of essential drugs and with 18.7% used for overall management of the program (its grants, monitoring and evaluation, reporting, documentation of best practices, technical and representational meetings, and advocacy activities).

**Figure 4.**
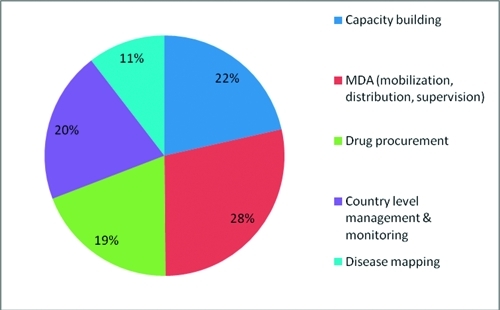
Distribution of expenditures by the Neglected Tropical Disease Control Program during its first three years. Of the $30.82 million expended on country program implementation during the first 3 years, 22% (dark blue) was spent on capacity building, 28% (red) on mass drug administrations (MDAs) (mobilization, distribution, and supervision), 19% (green) on procurement of non-donated drugs, 20% (purple) on country-level management and monitoring, and 11% (light blue) on disease mapping.

The breakdown of program expenditures reflects the diverse set of activities that must be supported to enable treatment of persons at the community level. Of the 81.3% of funds going directly for country program activities and supplies (Figure 4), the largest portion (28%) was spent on the MDAs themselves, including for social mobilization, drug distribution, and personnel supervision. Almost equal portions (19–22%) went to drug procurement, capacity building, and country-led management and program monitoring. The remaining 11% was required for the initial mapping to define disease distribution.

Although not yet specifically documented for this program, it should be noted that previous costing studies of programs to control one or more NTDs have shown that in addition to the external support received by disease-endemic countries, the national ministries contribute approximately an equal level of resources in staff salaries and in-kind resources to achieve success.^14^

## Discussion

The whole concept of program integration is, undeniably, complex and involves, as described by some,^15^ not only the multiple domains of policy, activity, and organizational structure, but also multiple levels of integration intensity: coordination, collaboration, and consolidation. To others, however, integration is more an attitude than a formula, a focus on trying always to find ways to carry out multiple activities with the most efficient and cost-effective use of available resources. From either perspective, the arguments for integrating control programs that target the NTDs remain strong: the populations affected are much the same, the strategic approach (preventive chemotherapy) is essentially identical, and the drugs for implementing programs are largely donated, readily available, highly safe, and effective. Furthermore, opportunities to embed or integrate NTD control activities within school-health programs and through other platforms of health service delivery offer the promise of greater efficiency, long-term sustainability, and national capacity strengthening.

Early pilot studies confirmed the general feasibility of successful integration of NTD control efforts,^6^–^8^ but two major questions have remained. 1) Can the integrated NTD control activities effective in pilot studies be successfully expanded to programs at full national scale? 2) Which elements of individual NTD programs are most successfully integrated and how are they best implemented? The findings in this present report address the first question, showing clearly that integration of multiple disease-specific NTD control programs can be successfully implemented at full national scale. Assessment and analyses addressing the second question, to define the most effective and cost efficient ways of integrating specific program activities, are still underway.

From the data in Figure 1, it is clear that funds from the USAID support of the NTD Control Program have been effective in facilitating national programs to support, organize, implement, and monitor integrated, formerly disease-specific programs targeting WHO's five tool-ready NTDs.^3^ In the 7 study countries receiving support during the program's first three years, progressive scaling up resulted in an additional 16 million persons receiving appropriate PCT in the first year, 27 million in the second year, and 55 million in the third year, for a cumulative total of more than 98 million persons reached in three years and quite clearly proving the feasibility for integrated NTD programs to be carried out at full national scale.

Introduction of this funding also effected a major qualitative change in national programs targeting the NTDs. Even though in the year prior to the NTD Control Program, up to 33 million people had received treatment of NTDs in disease-specific programs in the target countries, these national programs were constantly challenged to identify funds, most often on a yearly basis, to support their program activities. With the NTD Control Program, a secure funding source was established, so that these national programs not only could achieve broader disease control in their populations but also could undertake proper planning to address their NTD problems more effectively and cost efficiently.

Different from single-disease programs where each person reached equals one treatment given, in the integrated programs each person reached receives a drug ‘package' usually containing more than one drug. Therefore, the metric ‘treatments delivered,' the number of times a single drug dose is administered, had to be developed to record this programmatic achievement. More than 222 million treatments were provided by national programs during the first three years of the NTD Control Program. It can be predicted with certainty, based on recent studies assessing the impact of disease-specific NTD programs,^16^,^17^ that when the program assesses the health benefits from these treatments after 3–5 years of treatment, its impact on personal, societal, and economic well-being will be seen to be enormous.

What even these numbers by themselves fail to impart, however, is an understanding of the magnitude and importance of the public-private partnership between specific pharmaceutical companies and the public sector. It has been the donation of extraordinary amounts of drugs (by GlaxoSmithKline, Johnson & Johnson, Merck & Co., Inc., and Pfizer) that has made possible the successes of these global efforts to control or eliminate the NTDs. In addition, although the drug manufacturers have without question provided most of the drugs used in this program, the generic drugs needed for schistosomiasis and STH control and for LF elimination in countries outside Africa have also been obtained and provided to national programs in the NTD Control Program by a number of governmental and nongovernmental partner organizations, including the Department for International Development (United Kingdom), the United Nations Children's Fund, University of Notre Dame, WHO, the World Food Program, and World Vision.

In addition to its role in facilitating integrated national MDA programs to provide treatments for their at-risk populations, the NTD Control Program has, of necessity, supported countries in mapping the distribution of infection and developing, in collaboration with WHO, action plans that will enable them to begin or expand implementation of drug delivery in the coming years. Already the empirical experience gained from three years of program activities in the first seven implementing countries has contributed appreciably to the development of new guidelines, norms, and regionalized strategies that will facilitate and accelerate program activities in the many countries still needing to begin their integrated NTD control programs.

Major challenges still lie ahead for creating integrated programs targeting NTD control or elimination, not the least of which is the large number of countries requiring these programs. Such a need provides political challenges not just for the national governments, which must commit their limited resources and energy to the programs, but also for the bilateral donors to support these national commitments, the drug manufacturers to sustain their long-term pledges, and the various implementing partners necessary to support national programs to carry out their integrated NTD implementation strategies.

In addition to these political challenges, technical challenges also remain, the first being definition of the geographic scope of each of the NTDs. Mapping the infections must be completed, not just to identify prevalence, but, even more importantly, to define exactly what action must be taken at which level of prevalence and in which geographic area for each of the NTDs. Then, once it is determined what action is required for each of the NTDs, the treatment gaps for specific diseases (e.g., schistosomiasis [Figure 2]) must be addressed and the efficiencies of integration (in terms of both cost and impact) identified and quantified so that cost-efficient integrated programs can be established. It does, of course, remain absolutely essential that the specific goals of each individual program being integrated be met, including those program targets that are not MDA-dependent, such as morbidity control (surgical and otherwise) for LF and trachoma, and the water and sanitation goals of the schistosomiasis, STH, and trachoma programs. Sacrificing a program's goals simply for the sake of integration is totally inappropriate. Thus, the elimination targets for LF and blinding trachoma by 2020^11^,^12^ must be kept, and for onchocerciasis in the Americas and selected foci in Africa, the elimination targets must also be respected.^18^,^19^

In just three years, the NTD Control Program has exceeded program expectations that had been based on previous pilot program activities. This achievement demonstrates not only the feasibility of going to national scale with an integrated approach but also that efficiencies can be achieved through integration. These efficiencies come, in large part, from removing duplication in the operations of specialized disease programs as co-implementation is introduced. They also stem from the ability of the NTD programs to implement PCT through community networks, schools and existing health service delivery platforms, such as child health days. Going forward, even greater gains may accrue as opportunities are pursued strategically to leverage or complement other development-sector efforts, such as water and sanitation improvements, or other health-sector inputs, such as malaria control activities. This type of integration of NTD control within development efforts and on existing health-service delivery platforms holds promise for even greater program efficiency and its positive impact on strengthening national health systems. Although it is clear that not all integrated NTD program activities will have such health system strengthening effects,^20^ it is also clear that many of these integrated program activities can very definitely build stronger systems for delivering healthcare and disease prevention to these most underserved populations in NTD-endemic countries.^21^

Although the creation of integrated NTD control programs has brought with it a wide range of political and technical challenges whose importance should not be underestimated,^20^ there can be little question but that today's increased attentiveness and support for NTD control provide important opportunities to advance the health of the world's neediest populations towards global health equity in ways never before possible. This current experience of the USAID NTD Control Program has proven already that an integrated approach to these diseases is feasible at full national scale. What still remains now is to draw further on the experiences of this young program to define those elements of disease-specific program integration that can yield the greatest benefits cost-effectively and cost-efficiently. There appears to be no reason why such integrated NTD programs, following general WHO guidelines and the accumulating experience of a growing number of countries, cannot be replicated in all places where they are needed, so long as necessary political commitment and support can be maintained.

## Figures and Tables

**Table 1:** Disease-specific guidelines for neglected tropical diseases*

Disease	Diagnostic approach for mapping	Threshold for implementation of PCT interventions	Unit of implementation	At-risk population targeted	Drugs	Frequency of intervention†
Lymphatic filariasis (in countries where onchocerciasis is co-endemic)	Antigen detection (ICT) or microfilaria detection (microscopy) in whole blood	Prevalence ≥ 1% in adults in some part of an implementation unit	District or other as defined for ease of operation	≥ 5 years old	IVM and ALB	Once per year (anticipated 4–6 years)
Lymphatic filariasis (in countries where onchocerciasis is not co-endemic)				≥ 2 years old	DEC and ALB	
Onchocerciasis–APOC	Nodule detection using rapid techniques	Presence of palpable nodules ≥ 20% in adult men	Mesoendemic or hyperendemic focus (reflecting river basins)	≥ 5 years old	IVM	Once per year, except in special circumstances
Onchocerciasis–OEPA	Skin snip	Prevalence of infection ≥ 1% in an implementation unit	Endemic focus	≥ 5 years old	IVM	Twice per year (anticipated 10–14 years)
Schistosomiasis	Parasitologic methods 1) detecting eggs in urine or stool (microscopy) 2) detecting blood in urine (hemastix or questionnaires)	High risk: prevalence of infection ≥ 50% in SAC	District, sub-district, or community	SAC and adults	PZQ	Once per year
		Moderate-risk: prevalence of infection ≥ 10% but < 50% in SAC		SAC and at-risk adults		Once every two years
		Low-risk: prevalence of infection < 10% in SAC		SAC		Twice during primary schooling
Soil-transmitted helminthiasis (ascariasis, trichuriasis, hookworm)	Detecting eggs in stool (microscopy)	High-risk: Prevalence of any STH ≥ 50% in SAC	District, sub-district or community	SAC, preschool children, women of childbearing age, pregnant women in second and third trimesters, special adult populations	ALB or MBD	Twice per year
		Low-risk: Prevalence of any STH ≥ 20% and < 50% in SAC				Once per year
Trachoma (blinding)	Eyelid examination for follicular inflammation (TF)	TF prevalence ≥ 10% in 1–9 year-old children	District	Everyone ≥ 6 months old with azithromycin; Children <6 months with TET	AZT and TET	Once per year (AZT); twice per day for 6 weeks (TET)

* Consistent with established and currently followed World Health Organization recommendations^3^. PCT = preventive chemotherapy; ICT = immunochromatography; IVM = ivermectin; ALB = albendazole; DEC = diethylcarbamazine; SAC = school age children; STH = soil-transmitted helminths; PZQ = praziquantel; MBD = mebendazole; AZT = azithromycin; TET = tetracycline; TF = trachomatous inflammation; APOC = African Programme for Onchocerciasis Control; OEPA = Onchocerciasis Elimination Program in the Americas.

† Duration of intervention varies for each disease.

**Table 2:** Principal drug distribution strategy in disease-endemic districts*

NTD control program	Country	Principal drug distribution strategy in disease-endemic districts†					Lead NGO
		LF	Onchocerciasis	Schistosomiasis	STH	Trachoma	
“Fast-track” countries	Burkina Faso	Community	Community	Community	Community	Community	Schistosomiasis Control Initiative
		Household	Household	School-based	Household	Household	
		Health center	Health center	Household	Health center	Health center	
		Mobile	Mobile	Health center	Mobile	Mobile	
				Mobile			
	Ghana	Community	Community	School-based	Community	Transmission of blinding trachoma interrupted	World Vision
	Mali	Community	School-based	School-based	Community	School-based	Helen Keller International
		School-based	Household	Household	School-based	Household	
		Household		Mobile	Household	Mobile	
		Mobile		Mobile			
	Niger	School-based	NA	School-based	School-based	School-based	Schistosomiasis Control Initiative
		Household		Household	Household	Household	
	Uganda	Community	Community	Community	Community	Community	RTI International
		School-based	Household	School-based	School-based	School-based	
		Household		Household	Household	Household	
				Health center	Health center		
Additional countries	Haiti	School-based	NA	NA	School-based	NA	IMA-World Health
		Distribution			Distribution		
		posts			posts		
	Sierra Leone	Community	Community	School-based	Community	NA	Helen Keller International
		Household	Household		School-based		
					Household		
	Bangladesh						RTI International
	Cameroon						Helen Keller International
	Nepal						RTI International
	Southern Sudan						Malaria Consortium
	Togo						Health and Development International

* NTD = neglected tropical disease; LF = lymphatic filariasis; STH = soil-transmitted helminths; NGO = nongovernment organizations; NA = not applicable.

† General features of different distribution strategies described by national programs. Community distribution = in the market, mosque, or other busy places, common in urban settings; School-based distribution = in schools, targeting only children in schools; Household distribution = house-to-house, where the drug distributor brings the drugs to persons in their homes; Health center distribution = at a health center, where persons come to the health center to receive the drugs; Mobile distribution = through distributors traveling by vehicle to find households in remote areas, particularly in nomadic zones; Distribution posts = at locations such as schools, churches, or along the roadside, used in both rural and urban settings.

**Table 3:** Guidelines for disease-specific mapping

Disease	Guideline
Lymphatic filariasis	
Indicator	Prevalence of *Wuchereria bancrofti* antigenemia or *Brugia* microfilaremia
Persons tested	> 15 years old
	Living > 10 years in the community/village
Diagnostic tool	Immunochromatograpy (ICT) antigen test of finger stick blood or parasitologic examination of night blood films
Sample size	Up to 300 to identify at least 1 antigen-positive or microfilaremia-positive person (i.e., exceeding threshold of 1%)
Sampling frame	At least 1 village/site in an implementation unit
	Convenience sample or otherwise
Onchocerciasis	
Indicator	Prevalence of subcutaneous nodules or *Onchocerca volvulus* microfilariae in the skin
Persons tested	50 adults ≥ 20 years of age and living in the village for > 10 years
Diagnostic tool	Palpation of subcutaneous nodules (also possible:parasitologic examination of skin snip)
Sample size	50 per village; 2–4% of villages in focus
Sampling frame	Convenience or otherwise
Schistosomiasis	
Indicator	Questionnaire; prevalence of microhematuria or parasite eggs in urine for *Schistosoma haematobium*
	Prevalence of parasite eggs in stool for *S. mansoni*
Persons tested	School age children (7–14 years of age)
Diagnostic tool	Dipsticks for microhematuria/urine filtration for *S. haematobium*
	Kato-Katz or sedimentation test for *S. mansoni*
Sample size	50 school age children per school or site
Sampling frame	At least 5 villages with expected high prevalence in each ecologic zone
	In the village: convenience sample
Soil-transmitted helminths	
Indicator	Prevalence of eggs in stool
Persons tested	School age children (7–14 years of age)
Diagnostic tool	Kato-Katz
Sample size	50 SAC per school or site
Sampling frame	5 villages with expected high prevalence in each ecologic zone
	In the village: convenience sample
Trachoma	
Indicator	Prevalence of trachomatous inflammation (TF) and trichiasis (TT)
Persons tested	1–9 year-old children for trachomatous inflammation (TF)
	> 15 year-old children for TT
Diagnostic tool	Clinical examination of eyes
Sample size	50–100 children per cluster
Sampling frame	20 clusters per implementation unit (district or other) Probability Proportional to Estimated Size

**Table 4:** Mapping of districts in NTD Control Program countries*

Disease	Baseline before NTD Control Program Start		Districts Mapped with USAID Support	Districts Mapped with Other Support	No. remaining districts that need mapping at the end of year 3
	No. districts already mapped	No. districts needing NTD mapping			
LF	493	33	8	12	13
Onchocerciasis	379	147	0	143	4
Schistosomiasis	346	180	170	0	10
STH	356	170	170	0	0
Trachoma	423	103	68	24	11

* Aggregated total number of districts in the first seven implementing countries (identified in Table 2). NTD = neglected tropical disease; USAID = United States Agency for International Development; LF = lymphatic filariasis; STH = soil-transmitted helminths.

**Table 5:** NTD Control Program–supported treatments*

Drug	Year 1	Year 2	Year 3
IVM	12,049,342	15,551,089	43,945,901
DEC	0	0	2,111,826
ALB/MBD	13,263,152	20,221,501	51,906,980
PZQ	2,621,978	8,839,281	10,783,581
AZT/TET	8,881,685	13,417,513	19,106,346
Total	36,816,157	58,029,384	127,854,635

* IVM = ivermectin; DEC = diethylcarbamazine; ALB/MBD = albendazole/mebendazole; PZQ = praziquantel; AZT/TET = azithromycin/tetracycline.

**Table 6:** Number of tablets of donated drugs provided to national NTD programs in year 3 of the NTD Control Program*

Country	ALB	IVM	PZQ	DEC	Zithromax†	MBD	Tetracycline (tubes)‡	Total tablets§
Burkina Faso	11,862,300	33,913,000			8,553,600		158,642	54,487,542
Ghana	8,753,500	28,633,500	9,724,000		53,280	3,615,000		50,779,280
Haiti	6,933,600			22,300,000				29,233,600
Mali	4,976,900	14,494,500	3,000,000		8,972,640		198,904	31,642,944
Niger	8,465,000	22,128,500	5,498,500		11,509,920		200,000	47,801,920
Sierra Leone	4,500,000	16,716,850	3,000,000			3,797,498		28,014,348
South Sudan	324,500	9,215,000	3,000,000		505,440		2,400	13,047,340
Uganda	13,947,700	30,286,000			5,598,720	7,000,000		56,832,420
Total	59,763,500	155,387,350	24,222,500	22,300,000	35,193,600	14,412,498	559,946	311,839,394§

* Because donated drugs are provided to the countries in the year prior to their distribution, the number of drugs delivered (e.g., here in year 3) will not equal the number of treatments provided in the same year. Of the provided drugs, essentially all are used for treating the NTDs according to the national strategies (indicated in Table 2) and with coverage effectiveness approximated in Table 7. Any drugs unused in one year are applied to the requirements for treatment in the following year. NTD = neglected tropical disease; ALB = albendazole; IVM = ivermectin; PZQ = praziquantel; DEC = diethylcarbamazine; MBD = mebendazole.

† In addition, 629,616 bottles of pediatric oral suspension (~3 pediatric doses per bottle) were provided.

‡ Tetracycline ointment tubes are used at the rate of 2 tubes per child for a 6-week course of treatment.

§ Does not include bottles of Zithromax pediatric oral suspension or tubes of tetracycline ointment.

**Table 7:** Programmatic coverage in NTD Control Program countries*

NTD Control Program	Country	Year 1	Year 2	Year 3
Fast-track countries	Burkina Faso	82–86	79–97	89–100†
	Ghana	78–88		71–92
	Mali	69–100†	58–88	85–89
	Niger	91–99	73–88	78–93
	Uganda		57–97	62–97
Additional countries	Haiti			100†
	Sierra Leone			82–93

* Presented as a range across the different drug packages used in each country. NTD = neglected tropical disease.

† 100% values likely reflect incomplete census counts of the targeted population.
